# RBM7 subunit of the NEXT complex binds U-rich sequences and targets 3′-end extended forms of snRNAs

**DOI:** 10.1093/nar/gkv240

**Published:** 2015-04-06

**Authors:** Dominika Hrossova, Tomas Sikorsky, David Potesil, Marek Bartosovic, Josef Pasulka, Zbynek Zdrahal, Richard Stefl, Stepanka Vanacova

**Affiliations:** 1 CEITEC–Central European Institute of Technology, Masaryk University, Brno, 62500, Czech Republic; 2 National Centre for Biomolecular Research, Faculty of Science, Masaryk University, Brno, 62500, Czech Republic

## Abstract

The Nuclear Exosome Targeting (NEXT) complex is a key cofactor of the mammalian nuclear exosome in the removal of Promoter Upstream Transcripts (PROMPTs) and potentially aberrant forms of other noncoding RNAs, such as snRNAs. NEXT is composed of three subunits SKIV2L2, ZCCHC8 and RBM7. We have recently identified the NEXT complex in our screen for oligo(U) RNA-binding factors. Here, we demonstrate that NEXT displays preference for U-rich pyrimidine sequences and this RNA binding is mediated by the RNA recognition motif (RRM) of the RBM7 subunit. We solved the structure of RBM7 RRM and identified two phenylalanine residues that are critical for interaction with RNA. Furthermore, we showed that these residues are required for the NEXT interaction with snRNAs *in vivo*. Finally, we show that depletion of components of the NEXT complex alone or together with exosome nucleases resulted in the accumulation of mature as well as extended forms of snRNAs. Thus, our data suggest a new scenario in which the NEXT complex is involved in the surveillance of snRNAs and/or biogenesis of snRNPs.

## INTRODUCTION

The recent expansion of transcriptome-wide screens led to the identification of the complexity of the eukaryotic non-coding RNAome. The processing and stability of these RNAs undergoes strict monitoring by RNA surveillance machines. In yeast, one of the key factors in these processes is a conserved ten to eleven-subunit protein complex with 3′-5′ exoribonuclease activity, called the exosome (1,2). Several cofactors direct the exosome to specific RNA targets. The most prominent nuclear cofactors in budding yeast are the Trf4/5-Air1/2-Mtr4 Polyadenylating (TRAMP) and Nrd1-Nab3-Sen1 (NNS) complexes (3–6). The TRAMP is composed of the non-canonical poly(A)-polymerase (PAP) Trf4p or Trf5p, the zinc-knuckle (ZnK) protein Air1p or Air2p and the RNA helicase Mtr4p (4–7). TRAMP adds untemplated oligo(A) tail to the 3′ end of the RNA substrate which serves as a landing platform for the exosome. The NNS is formed by two RNA-binding proteins Nrd1p and Nab3p and an RNA helicase Sen1p (8). It is believed, that the NNS complex has a central role in RNA polymerase II (RNAP II) transcription termination of non-coding RNAs such as cryptic unstable transcripts (CUTs), or sn/snoRNAs (9–11). The NNS complex directly interacts with the terminator motifs in the nascent transcript as well as with the RNAPII via its C-terminal domain (CTD) (8,12–14). Subsequently, after termination the NNS complex interacts with the TRAMP-exosome and mediates 3′ end trimming and maturation or complete degradation of a substrate (3,11,15).

Two TRAMP-like exosome cofactors have been identified in mammalian cells; the human TRAMP complex and the Nuclear Exosome Targeting (NEXT) complex (16). Surprisingly, regardless of the presence of the human Sen1p homolog—Senataxin, attempts to identify homologs of Nrd1 and Nab3 were so far unsuccessful. However, despite the lack of any sequence homology, the human CBCA complex (cap-binding complex (CBC) interacting with ARS2) shows functional analogy with the yeast NNS (17,18). CBCA physically interacts with the NEXT complex forming a CBCN complex, which promotes transcription termination at snRNA loci, and in promoter-upstream regions expressing PROMPTs (17,19). However, the downregulation of NEXT components did not result in RNA Polymerase II termination defects on snRNA genes and PROMPTS, respectively, suggesting that NEXT may play a different role in ncRNA biology (17).

The NEXT complex is composed of hMTR4 (SKIV2L2), zinc knuckle protein ZCCHC8 and RNA binding protein RBM7 (16,20). Apart from NEXT involvement in PROMPT turnover, several lines of evidence have linked NEXT to spliceosomes. All three subunits have been initially co-purified with spliceosomes in a large-scale proteomics screen (21). RBM7 was shown to interact with two essential splicing factors, SAP145 and SRp20 (22) and SKIV2L2 with U4/U6.U5 tri-snRNP-associated proteins (23). Moreover, the RBM7 subunit has been recently shown to target U1 and U2 snRNAs (17).

Our previous work identified all three components of the NEXT complex specifically co-precipitated with U_30_ RNA (24). In this work, we characterize the RNA-binding and structural properties of the RBM7 subunit. We demonstrate the RBM7 preference for U-rich pyrimidine motifs. We present the solution structure of RBM7 RRM and map the residues involved in RNA binding. Moreover, we show that this RNA binding mode is important for targeting different forms of snRNAs *in vivo* and propose the role of NEXT in metabolism of snRNAs.

## MATERIALS AND METHODS

### Cell culture and manipulation

Mammalian cells (HEK293T-Rex, HeLa) were maintained in Dulbecco's modified Eagle's medium supplemented with 10% fetal calf serum at 37°C in the presence of 5% CO2. For transient transfections, cells were grown to 70% confluency, and plasmid DNA was transfected using TURBOFECT (Fermentas) following the manufacturer's instructions.

### Protein extracts preparation and cell fractionation

HEK293 cells were washed, re-suspended in buffer containing 10 mM HEPES pH 7.9, 1.5 mM MgCl_2_, 10 mM KCl, 2 mM DTT and 0.2 mM PMSF and incubated 10 min on ice. Cells were broken in a Dounce homogenizer and nuclei were pelleted by centrifugation at 400 g for 15 min at 4°C. The supernatant represented the cytoplasmic fraction. Nuclear pellet was then incubated with buffer containing 20 mM HEPES pH 7.9, 25% glycerol, 20 mM KCl, 1.5 mM MgCl_2_, 0.2 mM EDTA, 2 mM DTT and 0.2 mM PMSF. Next, the concentration of KCl was increased up to 1.2 M and extracts were incubated for additional 30 min at 4°C. The insoluble fraction was removed by centrifugation at 9000 g for 1 h at 4°C. The cytoplasmic and nuclear extracts were subsequently dialyzed to the buffer containing 20 mM HEPES pH 7.9, 20% glycerol, 20 mM KCl, 1.5 mM MgCl_2_, 0.2 mM EDTA, 2 mM DTT and 0.2 mM PMSF. After dialysis extracts were centrifuged for 30 min at 9000 g at 4°C, supernatant was aliquoted and frozen in liquid nitrogen.

### RNA-based protein precipitation

Affinity purifications were performed with biotin-labeled U_30_ RNA and a random 30 nt RNA, which served as a control for unspecific binding to RNA (5′ GAACAUAUUUCACCAACAUUAUACUGUGUC 3′), the expression of which has not been detected in mouse and human cells (our BLAST search).

To remove unspecific binders, the protein extracts were first pre-cleared using 100 μl of packed Streptavidin agarose (SAg) resin (Thermo Scientific), washed twice with Low salt buffer (LSB) (20 mM HEPES pH 8.0, 100 mM KCl, 10 mM MgCl_2_, 0.01% NP40, 1 mM DTT). Fresh SAg was pre-blocked in blocking buffer (LSB containing 1 μg/ml RNase-free BSA, 20 μg glycogen, 50 μg yeast total RNA) for 1 h at 4°C with slow rotation. One milliliter of blocking per 100 μl of packed SAg beads buffer was used. SAg were then washed twice with High salt buffer (HSB) (20 mM HEPES pH 8.0, 300 mM KCl, 10 mM MgCl_2_, 0.01% NP40, 1 mM DTT) and stored as 1:1 slurry (SAg beads : HSB).

To prepare SAg-RNA matrix, 40 μl of pre-blocked slurry of SAg beads was mixed with 5 volumes of HSB containing 10 μg biotinylated U_30_ RNA oligo (Sigma) and 50U of RNasin Plus RNase inhibitor (Promega) in HSB. The mixture was incubated for 5 h at 4°C with rotation. SAg beads were collected by 1 min centrifugation at 1500g and washed three times with 1 ml of HSB. For protein precipitation, 150 μl of pre-cleared protein extract was added and incubated for 1 h at 30°C with rotation. SAg beads were briefly collected by centrifugation at 1500g and washed three times with 1 ml of HSB. Bound proteins were eluted with 20 μl of 1x SDS loading buffer. Proteins were separated on 12% polyacrylamide gels and silver stained.

### Analysis of proteins by mass spectrometry

Protein samples were processed by filter-aided sample preparation (FASP) method (25,26). Proteins were alkylated, digested by trypsin on filter unit membrane and resulting peptides were eluted by ammonium bicarbonate. Peptide mixture was dried under vacuum and transferred using peptide extraction procedure to LC-MS vial containing PEG (27). Peptides were injected to LC-MS/MS system (RSLCnano connected to Orbitrap Elite; Thermo Fisher Scientific, Waltham, MA, USA) after concentration under vacuum. MS data were acquired in a data-dependent strategy selecting up to top 20 precursors based on precursor abundance in the survey scan (350–1700 m/z). Low-resolution CID MS/MS spectra were acquired in ion trap. The analysis of the mass spectrometric RAW data files was carried out using the Proteome Discoverer software (Thermo Fisher Scientific; version 1.3) with in-house Mascot (Matrixscience, London, UK; version 2.4.1) and Sequest search engines utilisation. Percolator was used for post-processing of search results. Peptides with false discovery rate (FDR; q-value) < 1%, rank 1 and with at least 6 amino acids were considered. Label-free quantification using protein area calculation in Proteome Discoverer was used (‘top 3 protein quantification’ (28)). See supplementary methods for details.

### RBM7 constructs preparation

The region encoding amino acids 6–94 of RBM7 RRM was amplified with primers RBM7rrm_HindIII_For and RBM7rrm_XhoI_Rev from cDNA prepared from HEK293T-Rex cells. The polymerase chain reaction (PCR) product was inserted into pET28b between HindIII and XhoI sites allowing expression of N-terminaly fused 6xHis-Smt3 tag. The sequence of the final clone was verified by sequencing. The mutant forms were prepared by site-directed mutagenesis (Invitrogen). See supplementary material for the list of oligonucleotides. RBM7 gene was amplified using primers RBM7_HindIII_ATG_For and RBM7_BamHI_nonstop_Rev from cDNA prepared from HEK293 cells. The PCR product was inserted to pcDNA5/FRT/TO allowing expression of 3 x C-terminal FLAG tag. The sequence of the final clone was verified by sequencing.

### Expression and purification of recombinant proteins

Recombinant RBM7 RRM was expressed and purified from BL21-DE3 RIPL strain of *E. coli*. Bacterial cells were grown at 37°C and protein expression was induced at OD_600_ 0.5 with 0.4 M IPTG at 30°C overnight. Cells were harvested by centrifugation and lysed by sonication in buffer containing 50 mM Tris pH 7.0, 200 mM NaCl, 2 mM 2-mercaptoethanol and 0.1% NP-40, 10 mM imidazole. Lysate was cleared by centrifugation (17000 g, 30 min, 4°C). Protein was purified by Ni-NTA chromatography. SMT3 tag was removed by proteolysis with Ulp1 protease. Recombinant RBM7 RRM was further purified by gel filtration on Superdex 200 column (GE Healthcare) in buffer containing 50 mM Tris pH 7.0, 200 mM NaCl, 2 mM 2-mercaptoethanol, 10 mM imidazole. The purified protein was subsequently concentrated on Centricon centrifugal filter device 10 000 MWCO to approx. 0.8–1 mM concentration.

### Fluorescence anisotropy measurements

The equilibrium binding of RBM7 RRM to RNA was characterized by fluorescence anisotropy. The RNA was labeled at 5′ end with fluorescein fluorophore. The fluorescein was excited at 488 nm and its emission was collected at 520 nm. The width of both excitation and emission monochromatic slits was varying from 9 to 14 nm depending on measured RNA sequence. Integration time was set to 3 s. All measurements were conducted on a FluoroMax-4 spectrofluorometer (Horiba Jobin-Yvon). The instrument was equipped with a heated cell holder with a Neslab RTE7 water bath (Thermo Scientific). The system was operated by FluorEssence software (version 2.5.3.0 and V3.5, Horiba Jobin-Yvon). All measurements were performed at 20°C in 50 mM Tris 7.0 buffer supplemented with 200 mM sodium chloride and 10 mM 2-mercaptoethanol (pH 8). Ten nanomoles of RNA (in volume of 1.4 ml) was titrated with increasing amounts of RBM7 RRM protein sample (in the same buffer). Each data point in plot is an average of three measurements. The data were analyzed using Gnuplot (version 4.4.3) and Xmgrace (version 5.1.16). The data were normalized for visualization purposes and the experimental isotherms were fit to a single-site binding model according to Heyduk and Lee using non-linear least squares regression.

### NMR measurements and spectra assignment

All nuclear magnetic resonance (NMR) spectra for the backbone and side-chain assignments ∼0.3 mM uniformly ^15^N,^13^C-labeled RBM7 in 20 mM Bis-Tris buffer (pH 7.0), 200 mM NaCl, 4 mM 2-mercaptoethanol (93% H_2_O/7% D_2_O) were recorded on 700 MHz NMR spectrometer equipped with a cryoprobe at a sample temperature of 293.15 K. The spectra were processed using NMRPipe package and the protein resonances were assigned manually using the Cara software (http://cara.nmr-software.org). The ^1^H, ^13^C and ^15^N chemical shifts of the RBM7 were assigned using standard triple resonance experiments. All distance constraints were derived from the three-dimensional ^15^N- and ^13^C-separated NOESYs (with mixing time of 90 ms) collected on a 950 MHz spectrometer.

### Structure calculations

Structure determinations of the RBM7 was performed with the NOE assignment algorithm implemented in the CYANA program (29). This automated NOE assignment procedure is a re-implementation of the former CANDID algorithm (30) on the basis of a probabilistic treatment of the NOE assignment. CYANA carries out automated assignment and distance calibration of NOE intensities, removal of meaningless restraints, structure calculation with torsion angle dynamics and automatic upper distance limit violation analysis. The resultant NOE cross-peak assignments were subsequently confirmed by visual inspection of the spectra. In the next step, CYANA-generated restraints were used for further refinement of 20 preliminary structures with AMBER 12 software. These calculations employed a modified version (AMBER ff99SB) of the force field described by (31)), and an explicit water solvent. Structural quality was assessed using PROCHECK (32) and VERIFY3D (33). Molecular graphics were generated using PyMOL (http://www.pymol.org).

### Preparation of HeLa stable cell line with inducible expression of FLAG-tagged RBM7

Semi-confluent Flp-In™ T-REx™ (Life Technologies) HeLa cells (grown on 10 cm dish) were transfected with 1.5 mg of pOG44 plasmid and 0.4 mg of constructs pcDNA5/FRT/TO/RBM7–3xC-Flag or pcDNA5/FRT/TO/F13A-3xC-Flag, respectively using TURBOFECT (Fermentas) following the manufacturer's instructions. Cells with stably integrated constructs were selected in the presence of hygromycin B and blasticidin according to manufacturers instructions. Cells were cultivated until visible colonies appeared on the plate. Several clones were picked and tested for the Zeocin sensitivity and expression of the protein.

### RNA immunoprecipitation

Cells were grown to 90% confluence, washed with ice cold PBS and lysed in lysis buffer (LB) containing 150 mM NaCl, 50 mM Tris pH 7.6, 1% Triton X-100, EDTA-free Complete Protease Inhibitor Cocktail (Roche), 1 mM DTT, RNase In (Promega). Lysates were sonicated 6 × 10 s at 7% amplitude, incubated with Turbo DNase (Fermentas) for 15 min at 37°C and cleared by centrifugation. Supernatants were subsequently pre-cleared with magnetic beads without antibodies for 1 h at 4°C. FLAG M2 Magnetic beads (Sigma) were incubated with 10 μg of yeast tRNA for 1 h at 4°C. The pre-cleared extracts were applied on the pre-blocked FLAG beads and incubated for 2 h at 4°C. Beads were washed three times with LB and the bound RNA was extracted with the TriPure reagent (Roche) according to manufacturer's instructions. The isolated RNA was treated with the Turbo DNase (Fermentas) and used as a template for cDNA synthesis by using Superscript III reverse transcriptase (Invitrogen) and random hexamer primer mix. The cDNA was subsequently analyzed by real-time PCR.

### siRNA-mediated knockdown of NEXT components

The day before transfections 5 × 10^4^ cells were seeded. siRNA were transfected using INTERFERin transfecting reagent (Polyplus transfections) at 40 nM final concentration, following the manufacturer instructions. The siRNA treatment was repeated after 48 h and cells were collected for further analysis after another 48 h. See supplementary material for the list of siRNAs used.

### Western blot analysis

The proteins were separated by SDS-PAGE and transferred to nitrocellulose membrane by semi-dry blotting (BioRad). The list of antibodies used in this study is in the supplementary material.

### RNA isolation, cDNA synthesis and quantitative PCR

Total RNA was isolated with TriPure isolation reagent (Roche) according to manufacturer's instructions, followed by RNase-free DNase (TURBO DNase, Fermentas) treatment. The total RNA concentration was measured in a Beckman Coulter DU 730. Two micrograms of purified RNA was reverse transcribed using random hexamers and SuperscriptRT III (Invitrogen) according to the manufacturer's instructions. Real-time quantitative PCR (RT-qPCR) was performed using FastStart Universal SYBR Green Master (Roche) and gene-specific primer pairs on Q-PCR Light Cycler 7500 (Applied Biosystems). Each experiment was performed in at least three biological replicates. Transcript abundance was calculated by the ΔΔCt (delta delta Ct) method. Data were normalized to an internal control of the housekeeping gene HPRT mRNA or 18S rRNA for snRNA detection. Results are expressed as means and standard errors of the mean. *P*-values were calculated by two-tailed paired *t*-test; *P*-values < 0.1 were considered significant.

## RESULTS

### The identification of oligo(U) RNA binding proteins in HEK293 cells

**Figure 1.**
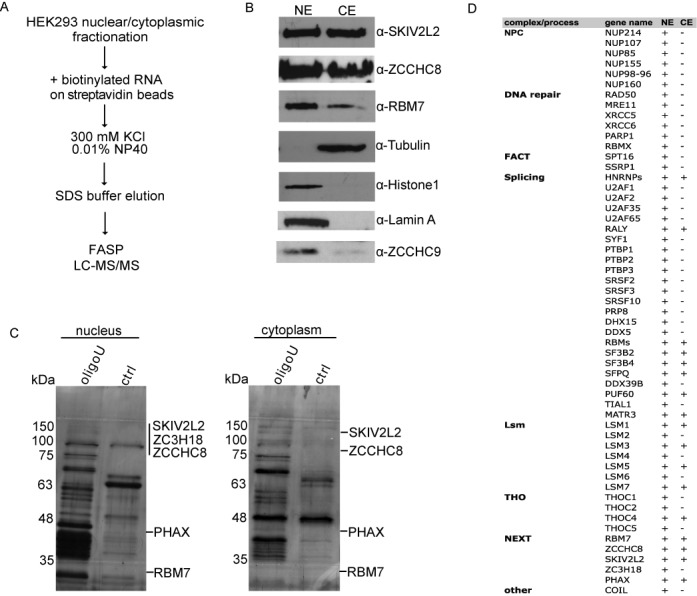
List of proteins bound to U_30_ RNA in HEK293 cells. (**A)** A schematic overview of the protocol. FASP stands for filter-aided sample preparation. (**B**) Western blot analysis of the cell fractionation. Tubulin was used as a cytoplasmic marker, and histone H1, lamin A and ZCCHC9 represented nuclear markers. (**C**) Protein profiles in control and U_30_ RNA-bound nuclear and cytoplasmic extracts. Equal amounts of eluates from control RNA (Ctrl) and U_30_ RNA bait fractions were separated on 12% SDS-PAGE gel and silver stained. (**D**) List of selected factors accumulated exclusively on the U_30_ RNA in human and mouse cells (24) in at least one replicate. The factors are grouped based on protein complexes they form. The last two columns display the presence (+) or absence (−) of particular protein in nuclear (NE) or cytoplasmic (CE) fraction, respectively. For full list of proteins see Supplementary Tables S1 and S2. NPC—nuclear pore complex, FACT—facilitates chromatin transcription/transactions complex, Lsm—SM-like genes forming U6 snRNP complex, THO—ribonucleoprotein nuclear export complex, NEXT—nuclear exosome targeting complex.

We have recently identified several proteins that were precipitated on oligo(U) RNA from mouse embryonic stem cell (mESC) extracts (24). We have noticed that RBM7, SKIV2L2 and ZCCHC8, which compose the NEXT complex, were present in nuclear fractions that were specifically bound to oligo(U) RNA and were absent in control RNA samples. To test if binding to oligo(U) is a general feature of NEXT, we performed RNA-based precipitations from other cell type, the Human Embryonic Kidney 293 (HEK293) cells. For that, we performed a crude cellular fractionation of HEK293 cells in order to enrich for nuclear and cytoplasmic proteins, respectively (Figure 1A and B). The efficiency of fractionation was monitored by western blot. Part of the nucleoplasmic proteins was also detected in the cytoplasmic fractions, which was probably due to the partial leakage of the nucleoplasm during the fractionation (Figure 1B). On the other hand, further investigations will be necessary to assess the possibility that NEXT localizes also to cytoplasm. We thus proceeded with both, nuclear as well as cytoplasmic fractions. The protein extracts were incubated with U_30_ and control RNAs (according to (24)), respectively, and proteins bound to particular RNA baits (Figure 1C) were analyzed by LC-MS/MS. The relative abundance of the identified proteins is shown in Supplementary Tables S1 and S2. In total, we have identified 58 proteins that showed at least five-fold enrichment in the U_30_ RNA samples in at least two biological replicates (Supplementary Tables S1 and S2) obtained from HEK293 cells. Importantly, 37 out of 58 oligo(U) bound proteins were also previously identified in U_30_ RNA pulldowns from mouse cells ((24), Supplementary Table S2). These 37 proteins included several factors and protein complexes already known to interact with poly(U) motifs or U-rich sequences, such as the nuclear and cytoplasmic Lsm complexes (34), PUF60 (35) or TIAR (36) (Supplementary Tables S1 and S2). In addition, we observed several proteins that have not been previously shown to bind U-rich sequences or to interact with RNA, respectively. The RNA-binding properties and significance of binding of several of these candidates is the subject of our following studies. Importantly, similar to our previous results with mouse cells (24), all three subunits of the NEXT complex were precipitated by U_30_ RNA also from HEK293 cells (Figure 1C and D, Supplementary Tables S1 and S2). The recent report from (17) has shown the close interaction between NEXT and ZC3H18 protein, which apparently bridges the NEXT to the CBCA complex. In agreement with that, the ZC3H18 protein was also detected specifically in oligo(U) precipitated extracts in our study (Figure 1C and D, Supplementary Tables S1 and S2). Furthermore, oligo(U) co-precipitated other nuclear RNA-processing complexes that may functionally interact with the NEXT; such as the PHAX, an RNP transport factor found in the CBCA co-purifications (17), the pre-mRNA splicing factors, components of the human nuclear-pore complex (NPC), the chromatin transcriptional elongation factor (FACT) and an mRNA export complex (THO). Surprisingly, factors involved in the non-homologous end-joining (NHEJ) double-strand break repair pathway RAD50, MRE11, XRCC5 and XRCC6 also accumulated significantly on the U_(30)_ matrix in at least one replicate (Figure 1D, Supplementary Table S1).

### RBM7 requires U-rich sequences for binding

**Figure 2.**
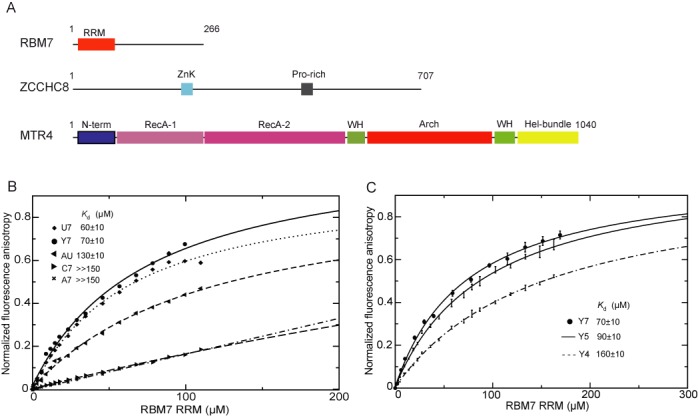
The RBM7 RRM domain exhibits specificity for U-containing oligopyrimidin sequences. (**A**) Schematic representation of the domain organization of NEXT complex subunits. Length of each protein in amino acids is indicated on the right. The boxes mark individual domains or protein regions; RRM, RNA recognition motif, ZnK, zinck knuckle, Pro-rich, proline-rich, N-term, N-terminal, RecA, helicase domain, WH, winged-helix domain, Hel-bundle, helical bundle. (**B**) Equilibrium binding of the RBM7 RRM with different fluorescently labeled RNA substrates monitored by fluorescence anisotropy. Binding isotherms and dissociation constants (*K*_d_) are shown for each substrate. (**C**) RBM7 RRM requires minimum 4 nt RNA for binding. Equilibrium binding of the RBM7 RRM with fluorescently labeled polypyrimidin RNA substrates of varying lengths monitored by fluorescence anisotropy. Binding isotherms and dissociation constants (*K*_d_) are shown for each substrate.

To uncover how the NEXT binds to oligo(U) RNA, we next asked which subunit of the NEXT complex is responsible for the oligo(U) RNA binding. We compared the domain organization of the three NEXT subunits (Figure 2A) to search for the oligo(U) binding subunit. SKIV2L2 is part of two distinct complexes, the NEXT and the TRAMP. We reasoned, that if SKIV2L2 was the main RNA binder, we would have initially co-precipitated subunits from both SKIV2L2-containing complexes in our oligo(U) pulldown. However, we observed subunits from NEXT only (Supplementary Tables S1 and S2). Moreover, SKIV2L2 is highly conserved across the eukaryotic kingdom (16) and its yeast homolog displays comparable affinities to both; oligo(A) and oligo(U) RNAs *in vitro* (37). ZCCHC8 possesses one zinc knuckle (ZnK) motif that can potentially interact with RNA, similarly to the yeast ZnK-containing protein Air2p (7). However, a single ZnK typically interacts with one or two nucleotides and is not able to recognize longer stretches of RNA (38,39). RBM7, the smallest subunit of NEXT, comprises an RNA recognition motif (RRM). Therefore, we hypothesized that the RBM7 RRM was the critical NEXT component responsible for recognition of oligo(U) stretches in RNA.

We subcloned, expressed and purified the RRM of RBM7, which contains fragment encompassing the region of amino acids 6 to 94 (Supplementary Figure S1A and B). To test the binding and substrate specificity of RBM7, we performed fluorescence anisotropy (FA) measurements with fluorescently labeled RNA. The RBM7 RRM revealed affinity (*K*_d_ = ≈60μM) for U-containing pyrimidine sequences, and lower affinity for the AU-rich heptamer (*K*_d_ = ≈130μM) (Figure 2B, Supplementary Table S3). In contrast, it failed to bind adenine and cytosine homoheptamers (Figure 2B, Supplementary Table S3). Moreover, RBM7 RRM required a minimum of four nucleotides long RNA for efficient binding (Figure 2C). We therefore propose that the NEXT complex is recruited to U-rich motifs via the RBM7 subunit.

### Solution structure determination of the RRM of RBM7

**Figure 3.**
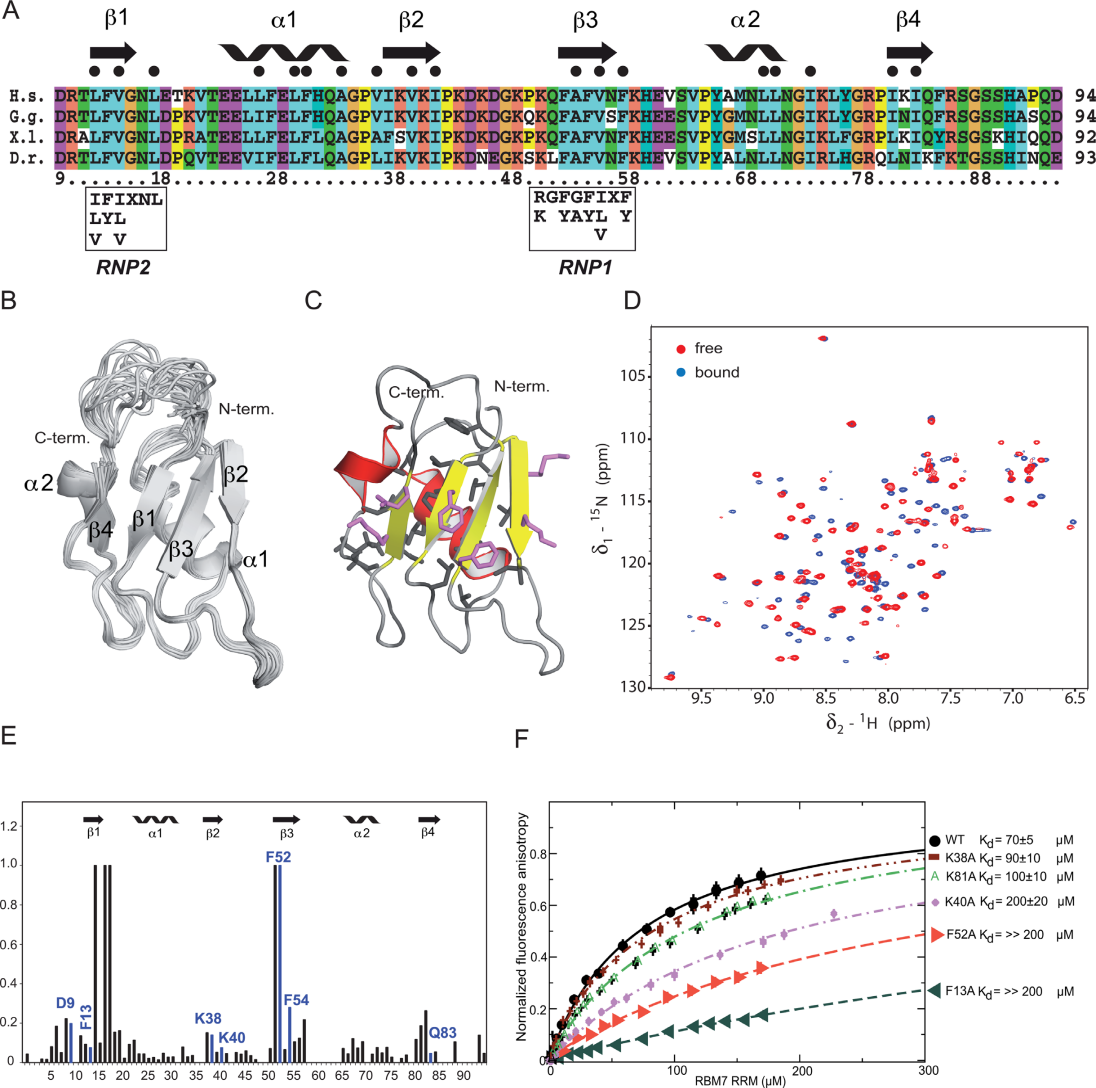
Structural studies and mapping of RNA-interacting residues of the RBM7 RRM. (**A**) Protein sequence alignment of RRM domains from different vertebrates species. Sequence alignment of RBM7 RRMs from four vertebrate species is depicted with its secondary structure elements and general consensus of RNP1 and RNP2 motifs. H.s. *Homo sapiens*, G.g. *Gallus gallus*, X.l. *Xenopus laevis*, D.r. *Danio rerio*. (**B**) Stereo view of the 20 lowest energy structures of RBM7 RRM. The protein backbone is shown as a wire model. (**C**) Stereo view of the representative (the lowest energy) structure of RBM7 RRM shown as a ribbon diagram. (**D**) ^1^H-^15^N HSQC spectra of RBM7 RRM alone (in red) and in the presence of 1 eq of 5′-CUCUCUC-3′ (in blue) at 303 K. (**E**) Quantification of chemical shift perturbations of RBM7 RRM upon binding to CUCUCUC RNA. The combined chemical shift perturbations ([ω_HN_Δδ_HN_]^2^ + [ω_N_Δδ_N_]^2^)^1/2^, where ω_HN_ = 1 and ω_N_ = 0.154 are weight factors of the nucleus (40), are plotted against the amino acid residue number. Large changes occur on the β-sheet surface. The residues with the chemical shift perturbations equal to one significantly moved at the beginning of titration in ^1^H-^15^N HSQC spectra but disappeared with additional RNA aliquots during the titration due to their unfavorable relaxation properties in the complex. Residues selected for site-directed mutagenesis are highlighted in blue. (**F**) Equilibrium binding of RBM7 RRM mutants with fluorescently labeled polypyrimidin RNA monitored by fluorescence anisotropy. Indicated RRM mutants of RBM7 along with the wild type of RBM7 RRM were titrated with fluorescently labeled CUCUCUC substrate. Binding isotherms and dissociation constants (*K*_d_) are shown for each mutant.

The RRM of human RBM7 is highly conserved among vertebrates (Figure 3A). In order to further analyze its RNA-binding properties, we solved the solution structure by NMR spectroscopy (Figure 3B and C). The three-dimensional structure of RBM7 RRM was determined by combined automated NOESY cross-peak assignment (40) and structure calculations with torsion angle dynamics implemented in the program CYANA 3.0 (29), followed by refinement in explicit solvent using AMBER 12 (41). An ensemble of the 20 lowest energy structures along with the representative lowest-energy structure are shown in Figure 3B and C, respectively. These structures have an average backbone root mean square deviation of 0.63 ± 0.08 Å for the secondary structure elements. A full summary of structural statistics including the backbone φ-ψ angle distribution is given in Table 1.

**Table 1.** NMR and refinement statistics for the RRM of RBM7

NMR distance and dihedral constraints	RBM7
Distance constraints	
Total NOE	1633
Intra-residue	453
Inter-residue	
Sequential (|i – j| = 1)	394
Medium-range (|i – j| < 4)	224
Long-range (|i – j| > 5)	562
Structure statistics^a^	
Violations (mean and SD)	
Number > 0.2 Å	0.20 (± 0.41)
Distance constraints (Å)	0.06 (± 0.052)
Max. distance constraint violation (Å)	0.21
Deviation from idealized geometry (ff99SB)	
Bond lengths (Å)	0.0023 (± 0.0001)
Bond angles (º)	2.57 (± 0.03)
Average pairwise r.m.s. deviation^b^ (Å)	
Heavy	0.63 (± 0.08)
Backbone	0.32 (± 0.06)
Z-score (Verify3D)	−2.09

^a^Calculated for an ensemble of the 20 lowest energy structures.

^b^Calculated for the structured part.

The overall structure of RBM7 RRM adopts a compact fold with a β1-α1-β2-β3-α2-β4 topology that is similar to the canonical fold of RRM family (42). The fold is composed of two α-helices that are packed along a face of a four-stranded antiparallel β-sheet. Interestingly, the α-helix 2 of RBM7 RRM is shorter that in the canonical RRM, as the sequence contains a conserved proline in position 64, which brakes the secondary structure of α2-helix and alters the arrangement of surrounding residues. The central hydrophobic core is composed of the residues shown in Figure 3A and C. RBM7 RRM contains a well-conserved signature of RRM family, the RNP1 and RNP2 sequences (Figure 3A) (43). These two conserved amino acid sequences found between positions 50–57 and 12–17 are located on the β3- and β1-strands, respectively. Their sequence compositions correspond to the general RNP2 and RNP1 consensus [ILV]-[FY]-[ILV]-X-N-L and [RK]-G-[FY]-[GA]-[FY]-[ILV]-X-[FY], respectively, except for the second aminoacid of the RNP1 (Figure 3A). RBM7 RRM has glutamine in the second position of RNP1 (Figure 3A). The presence of phenylalanines in RNP1 and RNP2 sequences, which usually mediate the stacking interactions with RNA bases, along with number of basic and polar residues on the β-sheet surface, suggests a potential role of RBM7 RRM in recognition of polypyrimidine (oligoY) stretches in RNA.

In order to identify regions that are involved in oligo(Y) RNA binding, we performed an NMR chemical shift perturbation study with CUCUCUC RNA. We titrated RBM7 RRM with CUCUCUC and used the ^1^H-^15^N HSQC experiment to monitor chemical shifts (Figure 3D). The backbone amide chemical shift perturbations resulting from complex formation can be seen in Figure 3E. Notably, there are four regions, which coincide with four β-strands that undergo significant chemical shift changes indicating RNA binding. The two α-helices are not involved in RNA binding. In order to identify critical amino acids involved in oligo(Y) binding, we chose solvent exposed conserved amino acids that displayed chemical shift upon RNA titration from each perturbed region (Figure 3A and E) and probed whether the RBM7 RRM binding is affected upon their mutation. The most prominent reduction in RNA binding was observed with F13A and F52A mutants (*K*_d_ >> 200μM), respectively (Supplementary Table S4). F13 is located on the surface of the first N-terminal β-strand, whereas F52 is positioned in the third β-strand (Figure 3C, E and F). There was also drop in affinity, albeit less pronounced, for the variants of basic residues, K40A (*K*_d_ ∼ 200 μM) and K81A (*K*_d_ ∼ 100 μM; Figure 3F). The residue K38 does not seem to have a key role in a substrate binding, because its mutation did not cause any significant drop of the *K*_d_ value (Figure 3F, Supplementary Table S4). In summary, these results indicated that the two conserved phenylalanine residues F13 and F52 are essential for direct contacts with oligo(Y) RNA.

### The NEXT complex targets snRNAs via RBM7 RRM *in vivo*

We next aimed to tackle the *in vivo* function of oligo(U) RNA binding by RBM7. Small nuclear RNAs, that were recently shown to be targeted by NEXT, contain encoded stretches of uridines close to their 3′ ends (44) as well as in transcription termination regions (16,17). Moreover, snRNAs have been shown to be post-transcriptionally uridylated at the 3′ ends in mouse and human cells (45). Therefore we next aimed to look, whether the RRM domain of RBM7 is responsible for snRNA recognition *in vivo*.

**Figure 4.**
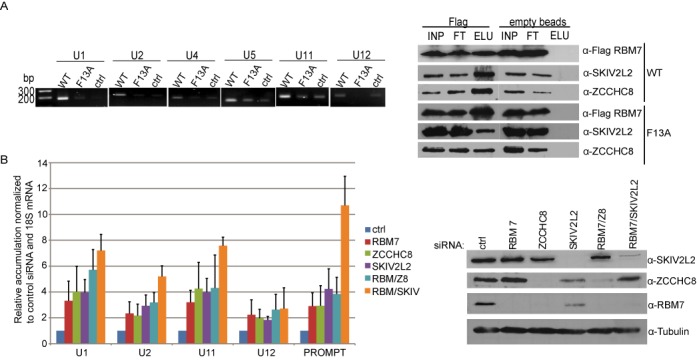
RBM7 mediates snRNA targeting by the NEXT *in vivo*. (**A**) RBM7 interacts with snRNAs *in vivo*. PCR amplification of cDNA prepared from RNAs coprecipitated with proteins indicated; agarose beads with no immobilized antibodies were used for background control. WT is Flag-RBM7, F13A is the Flag-tagged F13A mutant of RBM7. The efficiency of RBM7 and other NEXT subunits immunoprecipitation was monitored by western blot with specific antibodies shown on the right. Flag –anti-Flag antibodies immobilized on magnetic beads, empty beads – background control for unspecific binding to magnetic beads. INP whole cell lysate, FT unbound fraction, ELU bound fraction. (**B**) Downregulation of NEXT components leads to the accumulation of snRNAs. RT-PCR analysis of snRNAs in HeLa cells treated with siRNAs targeting individual subunits of the NEXT. ProSTK11IP PROMPT served as a positive control. Data are displayed as mean values normalized to the control siRNA and normalized to 18S rRNA as an internal control. Error bars, SD (*n* = 3 biological replicates). The efficiency of the knockdown was monitored by western blot shown on the right.

To test whether NEXT directly interacts with snRNAs, we performed RNA immunoprecipitation of RBM7-bound RNAs from HeLa cells stably expressing FLAG-tagged RBM7. We detected a significant enrichment of all tested snRNAs compared to the background signal obtained from cells not expressing tagged RBM7 (Figure 4A). Flag-RBM7 also co-precipitated both other NEXT subunits (Figure 4A), therefore we tested whether RBM7 is responsible for snRNA interaction. For that, we performed RIP with the RBM7 F13A mutant that showed defects in RNA binding *in vitro* (Figure 3F). The Flag-F13A mutant failed to precipitate snRNAs in amounts above the background control FLAG-GFP (Figure 4A). However, the F13A mutation did not appear to affect the NEXT complex assembly, because it also co-precipitated ZCCHC8 and SKIV2L2 (Figure 4A). Therefore we conclude, that RRM of RBM7 and not ZCCHC8 nor SKIV2L2 mediates the interaction of NEXT with snRNAs *in vivo*.

Next we assessed the steady state levels of selected snRNAs upon downregulation of the individual NEXT components. In agreement with the previous report (16), the ZCCHC8 knockdown caused simultaneous downregulation of RBM7. Furthermore, depletion of SKIV2L2 was accompanied by decreased levels of both ZCCHC8 and RBM7 (Figure 4B). The depletion of NEXT subunits individually resulted in two to four-fold accumulation of U1, U2, U11 and U12 snRNAs as well as of the ProSTK11IP PROMPT (Figure 4B). Co-depletion of RBM7 with SKIV2L2 (but not with ZCCHC8), revealed a synergistic increase of two-fold for snRNAs and five-fold for PROMPTs compared to the levels of the single knockdowns. In summary we showed that RRM of RBM7 is responsible for the recognition of oligo(Y) stretches *in vitro* and might recruit the whole NEXT complex to snRNAs *in vivo*.

### NEXT targets mature and extended forms of snRNAs

**Figure 5.**
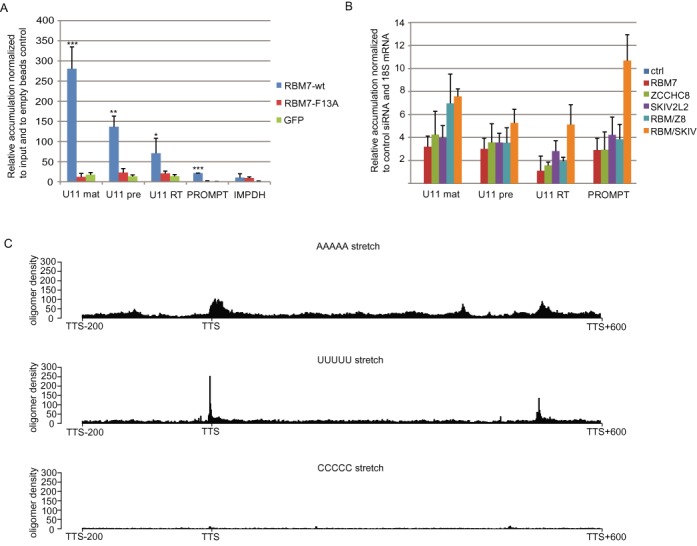
NEXT targets 3′-extended forms of snRNAs. (**A**) RBM7 co-precipitates different forms of snRNAs. RNAs bound to FLAG-RBM7 were reverse transcribed and quantified by RT-PCR by using probes to mature (mat), precursor (pre), and read-through (RT) regions of U11, and ProSTK11IP as a control. To monitor unspecific background precipitation of RNAs, we used agarose beads with no antibodies. Bar plot represents fold enrichments precipitated by using anti-Flag over control beads normalized to input. Error bars SD (*n* = 3 biological replicates), *P-value < 0.1, **P-value < 0.01, ***P-value < 0.001; *P*-values were calculated by two-tailed paired *t*-test. (**B**) Quantification of snRNAs by using probes as in A in cells treated with siRNAs targeting NEXT subunits. Data are displayed as mean values normalized to the control siRNA and normalized to the 18S rRNA as an internal control. Error bars, SD (*n* = 3 biological replicates). (C) Distribution of A_5_, U_5_ and C_5_ RNA motifs around the transcription termination site (TTS) of snRNAs. The plots reflect the regions with higher density of the pentamer motifs.

To gain insights about the role of NEXT in snRNA metabolism, we next asked which snRNA form is targeted by NEXT. For that, we designed a set of probes to amplify the following regions on snRNAs; the mature form, the precursor form, and the region downstream of the termination site (so called read-through), respectively (Supplementary Figure S2). The qPCR analysis of RNAs co-precipitated with the wild-type RBM7 showed 300-fold higher signal for mature U11 snRNA compared to the signal in the total RNA from the whole cell lysate (input for the IP), 150-fold enrichment for the precursor form and 70-fold enrichment for the read-through U11 snRNA (Figure 5A). The amount of pre-snRNA and RT-snRNA forms co-precipitated with the F13A RBM7 mutant was comparable to the background levels detected with the control GFP IP (Figure 5A).

The depletion of RBM7 and ZCCHC8 individually or in combination led to an increased signal for probes corresponding to mature and precursor regions of U1 and U11 snRNAs, but not significantly of their read-through forms (Figure 5B). On the other hand, SKIV2L2 knockdown cells exhibited significantly increased levels for probes for all three regions of U11 snRNAs (Figure 5B). We hypothesize, that at least part of the phenotype observed after SKIV2L2 depletion reflects defects in the function of the TRAMP rather than the NEXT complex. Based on these results, we conclude that the NEXT complex targets primarily mature and precursor forms of snRNAs.

Our *in vitro* RBM7-RNA binding assays revealed preference for minimum of four to five U-containing pyrimidine stretches. We performed a genome wide analysis of the oligo(U) occurrence in the proximity of snRNA genes. All non-overlapping snRNAs annotated in Ensembl gene model (46), release 74, data set ‘*Homo sapiens* genes’, version GRCh37.p13, were collected and analyzed (1916 records in total). We observed high frequency of occurrence of U_4_ and U_5_ stretches within the region up to 100 nt downstream of the snRNA mature 3′ end (Figure 5C, Supplementary Figure S3). Moreover, the U_4_ and U_5_ are the second most frequent four- and five-nucleotide long sequence motifs, respectively within 200 nt upstream and 600 nt downstream of the transcription termination site of snRNA genes (Supplementary Figure S3). We hypothesize, that these oligo(U) stretches could potentially serve as landing platforms for RBM7/NEXT binding. Interestingly, even higher occurrence of consequent identical nucleotides was observed for oligo(A) stretches at several positions downstream of the snRNA 3′ end (Figure 5C, Supplementary Figure S3), which could be putative binding sites for other factors. In contrary, plots for the stretches of cytosines were evenly distributed in the entire region around the transcription termination sites (TTS) (Figure 5C, Supplementary Figure S3).

Previous reports showed that depletion of NEXT components did not reveal any significant effect on Pol II occupation, and thus on transcription termination on snRNA genes (17). This is in agreement with our observation that RBM7 preferentially binds mature forms of snRNA *in vivo* (Figure 5A). Furthermore, depletion of RBM7 did not lead to the significant accumulation of RT forms of snRNAs (Figure 5B, Supplementary Figure S4). However, co-depletion of RBM7 and nuclear exosome components hDIS3 and hRRP6 led to the synergistic increase of signal for mature as well as extended forms of U1 and U11 snRNAs (Supplementary Figure S4). In contrary, the simultaneous depletion of SKIV2L2 and RRP6 did not display synergistic effect and in some cases it even suppressed the accumulation phenotype of the single SKIV2L2 knockdown (Supplementary Figure S4). We hypothesize that the phenotype observed with SKIV2L2 knockdown represents the role of SKIV2L2 as part of the TRAMP complex rather than the NEXT.

## DISCUSSION

The NEXT complex has been recently reported to be part of the CBCN complex, which includes ZC3H18, NEXT and CBCA complexes. Within this association, the NEXT bridges the CBCA interacting with the 5′ cap of a nascent or terminated transcript to the exosome (17). However, the principle of RNA target recognition by the NEXT complex remained unknown.

Here we demonstrate that the NEXT can be recruited to the RNA targets via its RBM7 subunit. The RRM of RBM7 binds U-containing pyrimidine stretches. Our structural studies show, that the U-rich polypyrimidine sequences are accommodated on the β-sheet of the RRM of RBM7 with canonical RRM fold. It is well established that the four-stranded β-sheet of the canonical RRM is the main protein surface involved in the interaction with the RNA and that it usually contacts two or three nucleotides (47–49). More nucleotides are usually recognized via additional N- and/or C-terminal extensions to the RRM (such as β-strand, α-helix, or loops) or via interplay of multiple RRMs (or RRM and another domains) (47–49). RBM7 contains only a single RRM and there are no N- and/or C-terminal extensions formed upon RNA binding, as evidenced by our NMR studies. Without the structure of RBM7-RNA complex, it is difficult to conclude how many polypyrimidines can be accommodated on the RRM β-sheet. The increasing binding affinity of longer RNA substrates likely reflects external cooperativity effects induced by the increased local concentration of polypyrimidines presented in the longer oligonucleotides (Figure 2), similarly as it was shown for the Tudor domain binding to peptides containing multiple methylation marks (50).

Our structural and functional analyses of RBM7 RRM show certain similarities with other polypyrimidine-binding proteins, albeit the general sequence alignments are rather poor (Supplementary Figure S1D). In general, the polypyrimidine recognition is usually achieved by a combination of two mechanisms: (i) selection on the basis of size (six-membered heterocyclic ring of polypyrimidines versus nine-membered heterocyclic fused-rings of purines) and (ii) recognition of the polypyrimidine unique pattern of hydrogen bond acceptors and donors. The recognition of polypyrimidine base edges can be achieved either by the RRM main-chain or side-chains, which determines the degree of specificity to some extent. The specific contacts mediated by long side-chains can vary as these residues are flexible and can rearrange upon binding to different ligands. Furthermore, these flexible side-chains often use water-mediated hydrogen bonds that can rearrange easily to accommodate different sequences. These structural strategies are used for example in the recognition of weak consensuses of relatively divergent polypyrimidine tract sequences that are important for splicing and alternative splicing (51–54). Interestingly, the RRM of RBM7 contains long-side chains (e.g. K38, K81 and K40) on the β-sheet of the RRM, suggesting the structural mechanism behind the specificity of RBM7 towards the polypyrimidine tracts. Purine substitutions in the polypyrimidine substrates resulted in weak binding that also confers shape-selective recognition mechanism involved in specificity of RBM7 to pyrimidine RNA sequences (Figure 2B). We cannot rule out the possibility that ZCCHC8 or SKIV2L2 also play a role in RNA recognition and binding. The previous work from Bernstein *et al*., revealed that Mtr4p, the yeast SKIV2L2 homolog possesses nanomolar affinity to oligo(A) and oligo(U) RNAs *in vitro* (37). Interestingly, the Mtr4p-U20 RNA-ATP is a less dynamic complex than when bound to A20 (37), suggesting, that the helicase is able to act on oligo(U) substrates. This supports the model, in which RBM7 specifically targets NEXT to U-rich pyrimidine regions and SKIV2L2 helicase activity helps to unwind secondary structures to promote subsequent digestion by nuclear exosome. RBM7 RRM does not bind oligo(A) RNA. It is possible, that ZCCHC8 and SKIV2L2 recognize other motifs on NEXT RNA targets. For instance, NEXT plays a role in PROMPT turnover. PROMPTs are polyadenylated (55), thus SKIV2L2 affinity to oligo(A) may be important in targeting PROMPTs.

High occurrence of U-stretches was identified within 1 kb of the PROMPTs or upstream of the transcription start sites (TSS) of mRNAs (55). This correlates with RBM7 binding to the same region more recently identified by RIP-ChIP analysis (17). All together, these results strongly suggest that NEXT is co-recruited to the PROMPTs also via direct interaction between RBM7 and U-rich regions within the RNA substrate. In addition to PROMPTs, our results indicate involvement of the NEXT complex in snRNA biology. Andersen *et al*. recently reported that RBM7 binds 3′ end extended U1 snRNAs and that co-depletion of ZCCHC8 and subunits from either exosome or CBCA results in accumulation of multiple extended forms of U2 snRNA (17). Our results extend the view on the NEXT function. Accumulations detected by QPCR implied, that RBM7 binds both, the mature, as well as 3′ extended forms of snRNAs. Because NEXT is apparently not involved in the transcription termination of snRNAs (17), the fact that it binds and targets their extended forms suggests its potential role in the snRNA surveillance and degradation. In light of the multiple evidence of the NEXT association with the splicing machinery in human cells (see above) it is possible that the NEXT can monitor snRNA quality during or after snRNP assembly, although, we cannot rule out other roles connected to the spliceosomes or splicing. At the moment it is also unclear at which step RBM7 binds to RNA. The physical association with CBCA, may co-transcriptionaly bring NEXT to its RNA targets. In the future, it will be interesting to look whether NEXT may be involved in spliceosomal regulation or even intron turnover. More importantly, due to its preference for oligo(U) stretches, it will be interesting to test, if NEXT may also target posttranscriptionally uridylated RNAs.

In summary, we showed that RRM domain of RBM7 protein is a potent binder of oligo(Y) stretches in RNA sequence *in vitro*. We further provide evidence that the RRM of RBM7 is responsible for recognition of mature as well as extended forms of snRNA *in vivo* and might guide the NEXT complex to oligo(U) containing RNA transcripts. We also observed synergistic effect of knockdown of RBM7 and exosomal nucleases RRP6 and DIS3 suggesting co-operation of the NEXT complex with the exosome. Taken together we propose RBM7 subunit of the NEXT complex to play a role in recognition of snRNAs and in the quality control and surveillance of snRNP biogenesis.

## ACCESSION CODES

The atomic coordinates for the NMR ensemble of the RNA-binding fragment of RBM7 have been deposited in the Protein Data Bank under accession code 2M8H.

## SUPPLEMENTARY DATA

Supplementary Data are available at NAR Online.

SUPPLEMENTARY DATA
