# Supplementary material for: RBM7 subunit of the NEXT complex binds U-rich sequences and targets 3′-end extended forms of snRNAs
Source: Nucleic Acids Res. 2015 Apr 6;43(8):4236–48. doi: 10.1093/nar/gkv240 (PMC4417160; doi:10.1093/nar/gkv240)
Supplement: SUPPLEMENTARY DATA [file supp_43_8_4236__index.html]

RBM7 subunit of the NEXT complex binds U-rich sequences and targets 3′-end extended forms of snRNAs — SUPPLEMENTARY DATA 

# RBM7 subunit of the NEXT complex binds U-rich sequences and targets 3′-end extended forms of snRNAs

## SUPPLEMENTARY DATA

**Files in this Data Supplement:**

- SUPPLEMENTARY DATA
- SUPPLEMENTARY DATA
- SUPPLEMENTARY DATA
