# Supplementary material for: RBM7 subunit of the NEXT complex binds U-rich sequences and targets 3′-end extended forms of snRNAs
Source: Nucleic Acids Res. 2015 Apr 6;43(8):4236–48. doi: 10.1093/nar/gkv240 (PMC4417160; doi:10.1093/nar/gkv240)
Supplement: SUPPLEMENTARY DATA [file supp_gkv240_nar-03007-y-2014-File007.docx]

**Title: RBM7 subunit of the NEXT complex binds U-rich sequences and targets 3′ end-extended forms of snRNAs**

**Authors:** Dominika Hrossova^1,2^, Tomas Sikorsky^1,2^, David Potesil^1^, Marek Bartosovic^1,2^, Josef Pasulka^1^, Zbynek Zdrahal^1^, Richard Stefl^1,2^*, Stepanka Vanacova^1^*

**Supplemental material and methods**

**
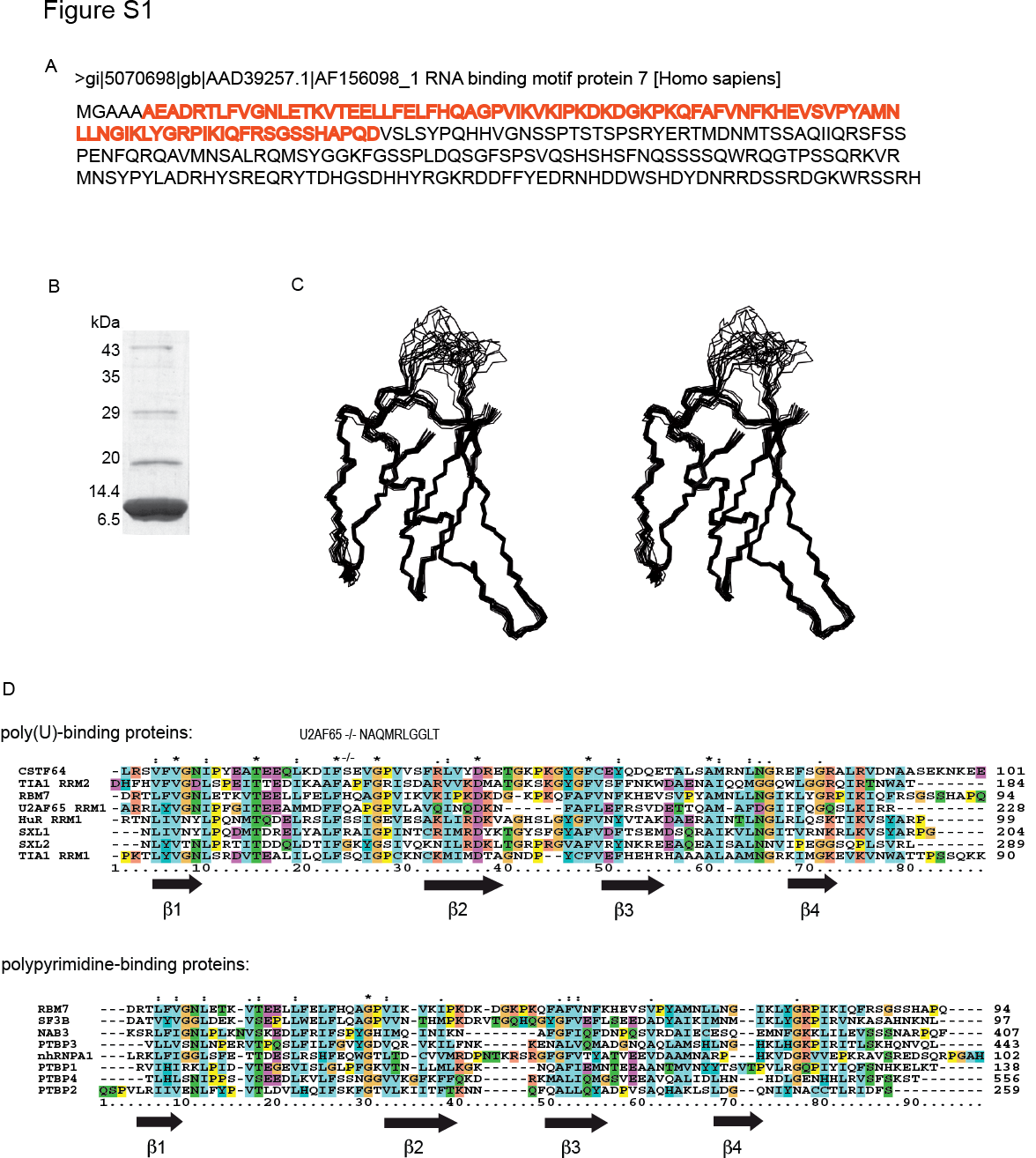
**

**
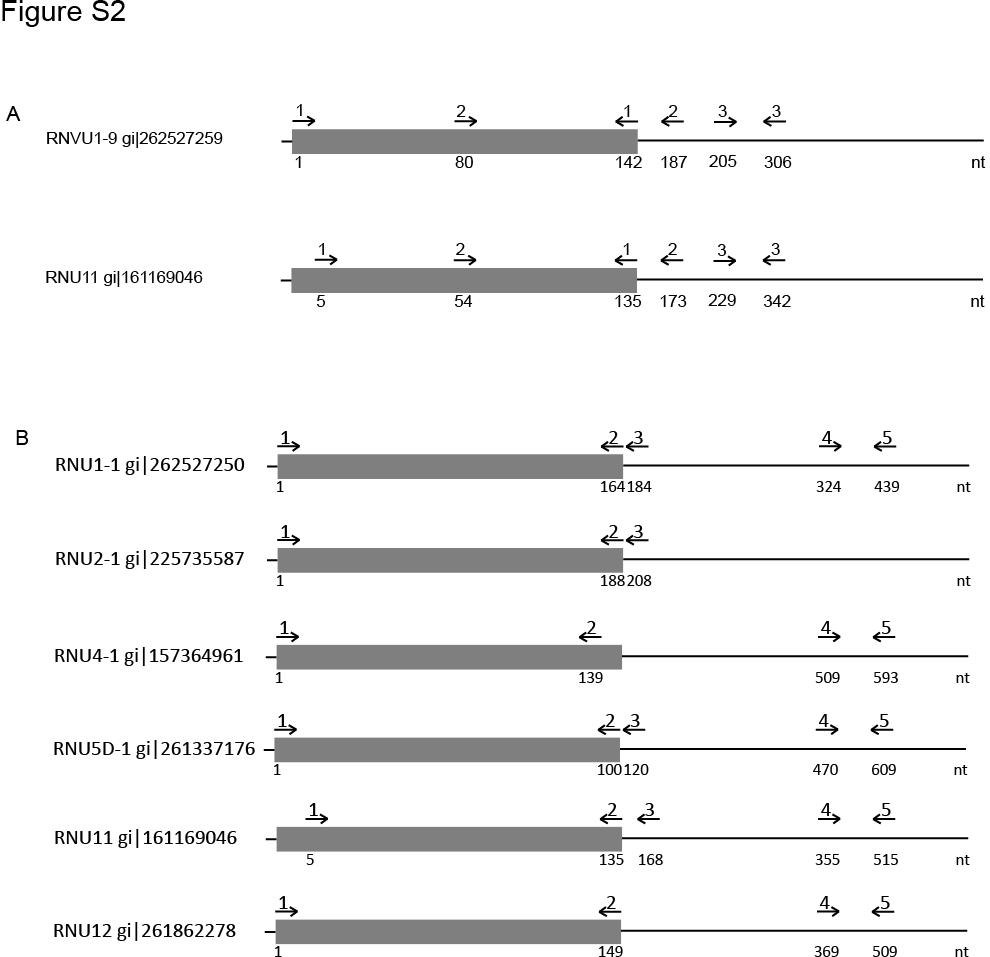

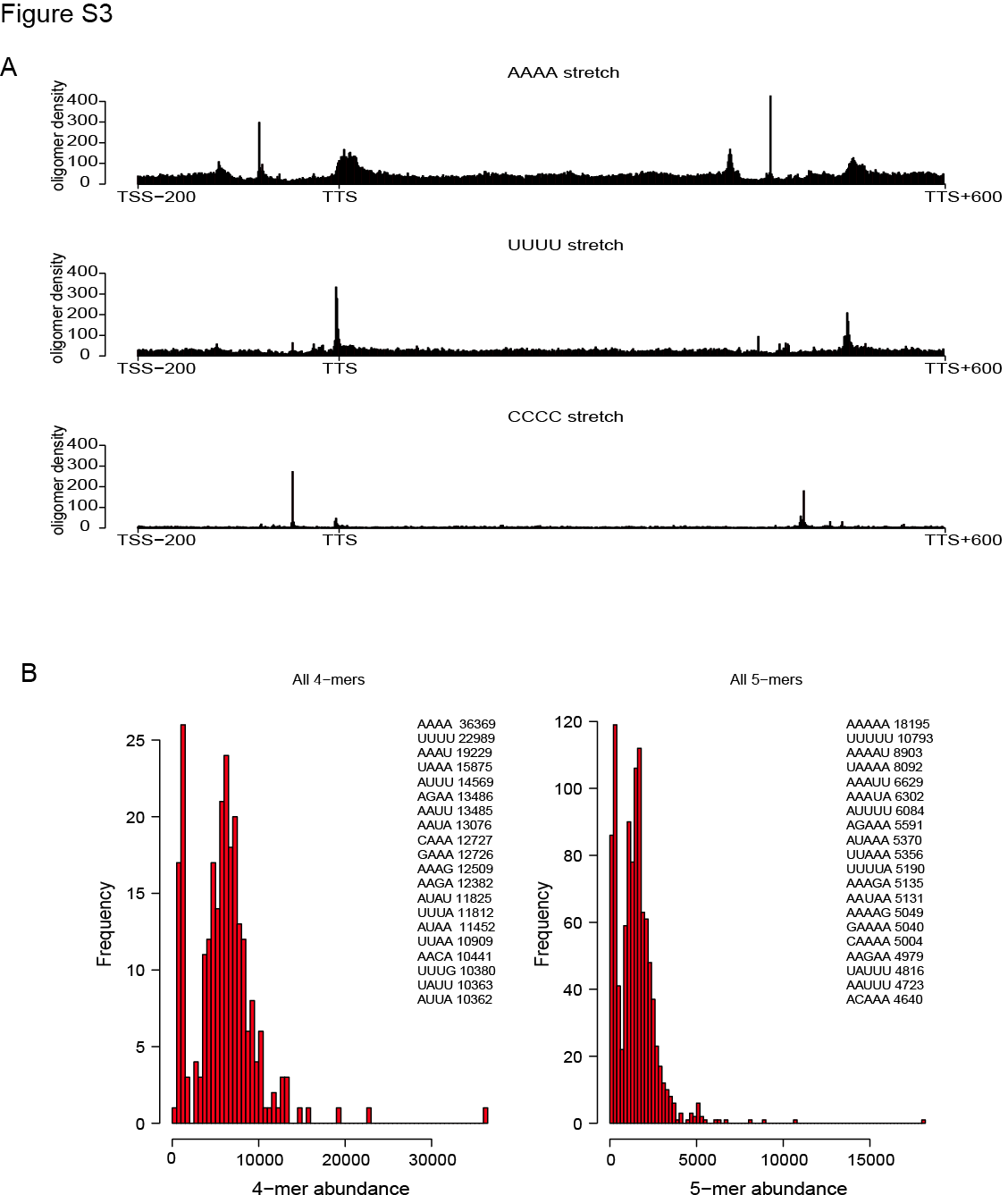
**

**
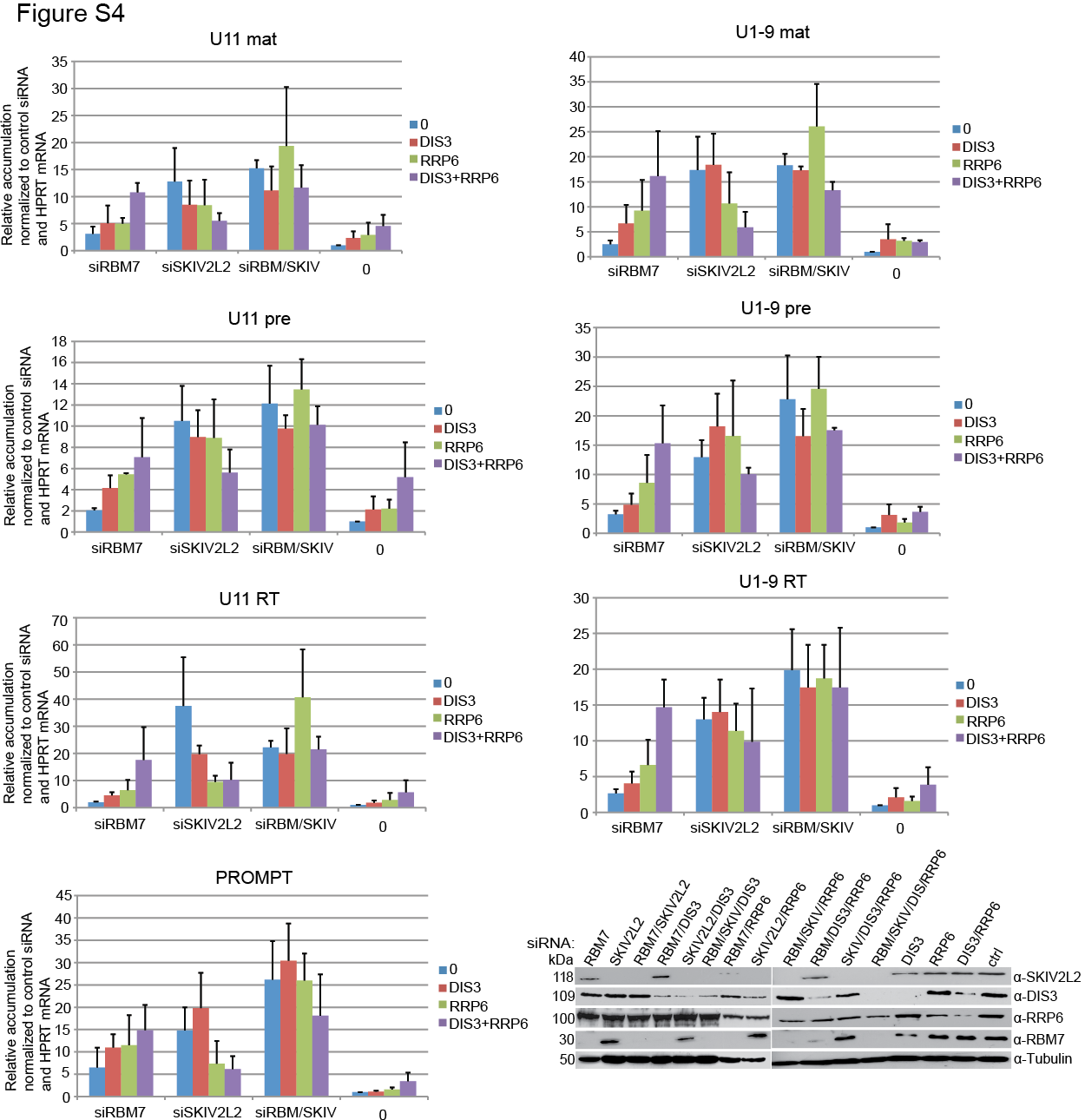
**

**Supplementary figure legends**

**Figure S1.** (A) Aminoacid sequence of RBM7 RRM is highlighted in red. (B) Coomassie stained PAGE gel showing purified recombinant RBM7 RRM that was used for NMR measurements. (C) Overlay of the 20 lowest-energy structures of the RBM7 RRM shown in stereo view. (D) Alignments of RBM7 RRM amino acid sequence with RRMs of known poly(U)-binding (upper) and poly(Y)-binding (lower) proteins.

**Figure S2.** Schematic view of RT-qPCR amplicon positions on snRNA genes. (A) Positions of oligonucleotides used in RIP and QPCR experiments connected to figure 5 and S3. (B) Positions of oligonucleotides used in RIP and QPCR experiments connected to figure 4 and 5. 1 – oligo pair for matured sequence amplification, 2 – oligo pair for precursor sequence amplification, 3 – oligo pair for readthrough sequence amplification.

**Figure S3.** (A) Distribution of A_4_, U_4_ and C_4_ RNA motifs around the transcription termination site (TTS) of snRNAs. The plots reflect the regions with higher density of the tetramer motifs. The sharp peak of U_4_ density in the TTS region reveals the increased occurrence of that motif in the 5 nucleotides immediately preceding the TTS. (B) Histogram of tetramer and pentamer abundance in transcribed sequences of all snRNAs (1916 entries) and their downstream regions (600 nt downstream of TTS). The plots display the abundance distribution of all possible tetra- and pentamers. The twenty most frequent motifs are listed on the right sides of the graphs.

**Figure S4.** The role of NEXT and exosome complexes in snRNA metabolism *in vivo.* (A-D) Increased levels of different forms of snRNAs upon depletion of the NEXT complex together with hDIS3 and hRRP6. RT-qPCR analysis of total RNA from HeLa cells treated with indicated siRNAs, using amplicons for mature (mat), precursor (pre), or read-through (RT) of U11, U1-9 snRNA and ProSTK11IP regions, respectively. Data are displayed as mean values normalized to the control siRNA (Dharmacon) and normalized to HPRT mRNA as an internal control. Error bars, s.d. (n=3 biological replicates). (E) Control western blot analysis of the NEXT and exosome subunits depletion in HeLa cells.

**Supplementary Tables**

**Table S1.** List of U_30_-enriched proteins identified by LC-MS in the nuclear fraction. Fold changes show ratio of the respective protein areas in each experiment. Ratio 100 means that the particular protein was identified with U_30_ RNA probe (U), but not with the control 30nt RNA (R). Ratio 0.01 means that a protein was identified in R but not in U sample; the sign "-" stands for a situation when a protein was not identified in any of the above.

**Table S2.** List of U_30_-enriched proteins identified by LC-MS in the cytoplasmic fraction.

**Table S3.** Binding affinities of WT RBM7 RRM to different RNA substrates

| **RNA substrate** | **5' to 3' sequence** | **Kd [μM]** |
| --- | --- | --- |
| U7 | UUUUUUU | 60±10 |
| Y7 | CUCUCUC | 70±10 |
| Y5 | CUUCU | 90±10 |
| Y4 | CUCU | 160±10 |
| AU7 | AUUAUUA | 130±10 |
| C7 | CCCCCCC | >>150 |
| A7 | AAAAAAA | >>150 |

**Table S4.** Binding affinities of RBM7 RRM mutants to Y7 RNA

| **RRM mutant** | **Kd [μM]** |
| --- | --- |
| K38A | 90±10 |
| K81A | 100±10 |
| K40A | 200±20 |
| F52A | >>200 |
| F13A | >>200 |

**Table S5.** List of RNA sequences used for pulldown experiments and FA measurements. Btn and Flc stand for biotin and fluorescein labels, respectively.

| **name of RNA** | **5' to 3' sequence** |
| --- | --- |
| Btn-Oligo U_30_ | Btn-UUUUUUUUUUUUUUUUUUUUUUUUUUUUUU |
| Btn-Random RNA | Btn-GAACAUAUUUCACCAACAUUAUACUGUGUC |
| Oligo A_30_ | AAAAAAAAAAAAAAAAAAAAAAAAAAAAAA |
| Y7 | Flc-CUCUCUC |
| U7 | Flc-UUUUUUU |
| C7 | Flc-CCCCCCC |
| A7 | Flc-AAAAAAA |
| AUre7 | Flc-AUUAUUA |
| Y4 | Flc-CUCU |
| Y5 | Flc-CUUCU |

**Table S6.** List of DNA oligonucleotides

| **DNA oligonucleotide** | **5' to 3' sequence** |
| --- | --- |
| RBM7_HindIII_For | CAACAAGCTTTGGGGGCGGCGGCGGCGGAAGC |
| RBM7_XhoI_Rev | ATCTCGAGTTAGTGTCGAGATGAGCGCC |
| RBM7rrm_HindIII_For | CAACAAGCTTTGGCGGAAGCGGATCGCACTCT |
| RBM7rrm_XhoI_Rev | ATCTCGAGTTAATCTTGTGGGGCATGACTAC |
| RBM7_HindIII_ATG_For | ACAAGCTTATGGGGGCGGCGGCGGCGGAAGC |
| RBM7_BamHI_nonstop_Rev | ATGGATCCATGTGTCGAGATGAGCGCC |
| RBM7F52A_f | AGGATGGTAAACCAAAGCAGGCTGCGTTTGTGAATTTCAAAC |
| RBM7F52A_r | GTTTGAAATTCACAAACGCAGCCTGCTTTGGTTTACCATCCT |
| RBM7K81A_f | AACTTTATGGAAGGCCTATCGCAATTCAATTTAGATCAGGAA |
| RBM7K81A_r | TTCCTGATCTAAATTGAATTGCGATAGGCCTTCCATAAAGTT |
| RBM7F13A_f | CGGAAGCGGATCGCACTCTCGCTGTGGGCAACCTTGAAACGA |
| RBM7F13A_r | TCGTTTCAAGGTTGCCCACAGCGAGAGTGCGATCCGCTTCCG |
| RBM7K38A_f | ACCAGGCTGGGCCAGTAATAGCGGTGAAAATTCCAAAAGATA |
| RBM7K38A_r | TATCTTTTGGAATTTTCACCGCTATTACTGGCCCAGCCTGGT |
| RBM7K40A_f | CTGGGCCAGTAATAAAGGTGGCAATTCCAAAAGATAAGGATG |
| RBM7K40A_r | CATCCTTATCTTTTGGAATTGCCACCTTTATTACTGGCCCAG |
| RBM7Q83A_f | ATGGAAGGCCTATCAAAATTGCATTTAGATCAGGAAGTAGTC |
| RBM7Q83A_r | GACTACTTCCTGATCTAAATGCAATTTTGATAGGCCTTCCAT |
| U1_fwd | ATACTTACCTGGCAGGGGAG |
| U1_rev | CAGGGGAAAGCGCGAACGCA |
| U1ext3’box_rev | CTTGGCGTACAGTCTGTTTTTGAAACTC |
| U1readthr_f | TCATAGGAGAAAAAGCGAGC |
| hsU1readthrREV3 | CCTCCGTGCATATGCGCTAG |
| U2_fwd | ATCGCTTCTCGGCCTTTTGG |
| U2_rev | GGGTGCACCGTTCCTGGAGG |
| hsU2ext_rev1 | AACACGTTGTACCCCGGAGG |
| U4_fwd | AGCTTTGCGCAGTGGCAGTA |
| U4_rev | TCCGTAGAGACTGTCAAAAA |
| U4-1_readthr_F | CTTTGCCGAACCCCTGTTTA |
| U4-1_readthr_R | CAAGAAAAGTCAACACCGC |
| hsU5D_fwd | GCTCTGGTTTCTCTTCAAAT |
| hsU5D_rev | AGGGCTTCAAAAAATTTGCT |
| U5D_readthr_F | GCCACCGTGCGAAAATTTGG |
| U5D_readthr_R | GGTGGATCACTTGAGGCCAG |
| hsU11_fwd | AGGGCTTCTGTCGTGAGTGG |
| hsU11_rev | AAAGGGCGCCGGGACCAACG |
| hsU11ext_rev | GCGTAATATTTCGCCTAACA |
| hsU11readthrFWD | TGTTCTCGTGTGGGAGGCTA |
| hsU11readthrREV2 | GGGGGGCTGAGGCAGGTCTC |
| hsU12_fwd | TGCCTTAAACTTATGAGTAA |
| hsU12_rev | CGGGCAGATCGCAACTCCCA |
| hsU12readthrFWD | CACAGTATTGACGTTGGGA |
| hsU12readthrREV2 | ATATTAAACGCTGTCACGAA |
| 18S_F | TTGTTGGTTTTCGGAACTGAG |
| 18S_R | GCAAATGCTTCGGCTCTGGTC |
| ProSTK11IP_fwd | GGGAGTCTAAGGAAAAGGAG |
| ProSTK11IP_rev | CAGTGAAAGGAGAGCGTATC |
| U5F-1matur_fwd | ATCTCTGGTTTCTCTTCATA |
| U5F-1matur_rev | CAGCCTTGCCTAGGCAAGAC |
| U5F-1precursor_f | TTCCGTGGAGAAAAACAACTATG |
| U5F-1precursor_r | AACACCCCACCCACACGTCTCT |
| U5F-1readthrough_f | AGGTTGGGGTAGCTCCACAAATGT |
| U5F-1readthrough_r | CTTATGAGGCTGAGGTGGGAGGAT |
| U1_9matur_fwd | ATACTTACCTGGCAGGGGAG |
| U1_9matur_rev | CAGGGGAAAGCGCGGACGCA |
| U1-9 precursor_f | AAATGTGGGAAACTCGACTGCATA |
| U1-9 precursor_r | AAGAATAACCCTTATAGGGGAGTC |
| U1-9 readthrough_f | GCTTGGGTGGCATGTTAAGTGTTC |
| U1-9 readthrough_r | ACTTAGTGTTAAAAGAGCTCACGG |
| U11 precursor_f | TGCGGAATCGACATCAAGAGATTT |
| U11 precursor_r | GTTACAAAAAGCACCACTTACTCC |
| U11 readthrough_f | GTATGAACCGCAGGTGACCTAA |
| U11 readthrough_r | TTGCTTAAGCTAGCCTCCCACAC |
| IMPDH_fwd | GTCCATGGCCTGCACTCT |
| IMPDH_rev | GTGGACACTGGGGTGCAT |
| HPRT_fwd | TGACACTGGCAAAACAATGCA |
| HPRT_rev | GGTCCTTTTCACCAGCAAGCT |

**Table S7.** List of siRNAs

| Target gene | sense strand: | anti-sense strand: |  |
| --- | --- | --- | --- |
| RBM7 (1) | gcguaaagucagaaugaautt | auucauucugacuuuacgctt |  |
| ZCCHC8 (1) | ggaauguaccucaggauaatt | uuauccugagguacauucctt |  |
| SKIV2L2 (1) | caauuaaggcucugaguaatt | uuacucagagccuuaauugtt |  |
| RRP6 | ggaugaguccuaccuugaatt | uucaagguaggacucauccgt |  |
| DIS3 | cccucgaauucgcauagaatt | uucuaugcgaauucgagggat |  |

**Table S8.** List of antibodies

| Antibody | target protein | Working dilution | produced in | origin |
| --- | --- | --- | --- | --- |
| ab70551 | SKIV2L2 | 1:5000 | rabbit | Abcam |
| ab83058 | Histone H1 | 1:1000 - 1:2000 | rabbit | Abcam |
| Monoclonal Anti-α-Tubulin | α subunit of Tubulin | 1:1000 | mouse | Sigma |
| (HPA013993) | RBM7 | 1:1000 | rabbit | Sigma |
| anti ZCCHC8 | ZCCHC8 | 1:1000 | rabbit | Sigma |
| anti hRRP6 | hRRP6 | 1:1000 | rabbit | Santa Cruz |
| anti hDIS3 | hDIS3 | 1:1000 | mouse | Abnova |
| anti Lamin A | anti Lamin A | 1:500 | rabbit | Santa Cruz |
| anti ZCCHC9 | anti ZCCHC9 | 1:500 | rabbit | (2) |

**Supplementary methods**

**FASP processing**

Protein pull-downs were processed by filter-aided sample preparation (FASP) method (3,4). The whole samples were mixed with 8M UA buffer (8M urea in 100 mM Tris-HCl, pH 8.5), loaded onto the Vivacon 500 device with MWCO 10 kDa (Sartorius Stedim Biotech) and centrifuged at 14,000 × *g* for 30 min at 20°C. The retained proteins were washed with 400 μL UA buffer. The final protein concentrates kept in the Vivacon 500 device were mixed with 100 μL of UA buffer containing 50 mM dithiothreitol and incubated for 30 min. After additional centrifugation, the samples were mixed with 100 μL of UA buffer containing 50 mM iodoacetamide and incubated in the dark for 30 min. After the next centrifugation step, the samples were washed three times with 400 μL UA buffer and three times with 200 μL of 50 mM NaHCO_3_. Trypsin (sequencing grade, Promega) was added onto the filter and the mixture was incubated for 14 h at 37°C. The tryptic peptides were finally eluted by centrifugation followed by two additional elutions with 50 μL of 50mM NaHCO_3_. Six Bovine Tryptic Digest Equal Molar Mix (Michrom Bioresources, Auburn, CA; proteins with annotation tag “B6E” in the list of identified proteins) was added to verify LC-MS quantification quality. Peptide mixture was dried under vacuum. Dried peptides in FASP tube were resuspended in 50 µl of 50% acetonitrile (with 2.5% formic acid) and transferred to LC-MS vial with already added polyethylene glycol (PEG; 2.5 µl of 0.01% PEG) (5). The FASP tube was washed again using the same solution (50 µl) and subsequently twice with 100% acetonitrile (2× 100 µl). Combined solution was concentrated under vacuum to volume below 25 µl. Formic acid (0.5 µl of 50% solution) and water (amount selected according to weight of peptide solution after concentration) was used to get 25 µg of peptide solution.

**LC-MS/MS analysis of peptides from FASP**

LC-MS/MS analyses of peptide mixture were done using RSLCnano system connected to Orbitrap Elite hybrid spectrometer (Thermo Fisher Scientific, Waltham, MA, USA). Prior to LC separation, tryptic digests were online concentrated and desalted using trapping column (100 μm × 30 mm) filled with 3.5-μm X-Bridge BEH 130 C18 sorbent (Waters, Milford, MA, USA). After washing of trapping column with 0.1% FA, the peptides were eluted (flow 300 nl/min) from the trapping column onto a Acclaim Pepmap100 C18 column (2 µm particles, 75 μm × 250 mm; Thermo Fisher Scientific, Waltham, MA, USA) by the following gradient program (mobile phase A: 0.1% FA in water; mobile phase B: 0.1% FA in acetonitrile): the gradient elution started at 1% of mobile phase B and increased from 1% to 45% during the first 40 min (28% in the 30^th^ and 45% in 40^th^ min), then increased linearly to 95% of mobile phase B in the next 2 min and remained at this state for the next 13 min. Equilibration of the trapping column and the column was done prior to sample injection to sample loop. The analytical column outlet was directly connected to the Nanospray Flex Ion Source (Thermo Fisher Scientific, Waltham, MA, USA).

MS data were acquired in a data-dependent strategy selecting up to top 20 precursors based on precursor abundance in the survey scan (350-1700 m/z). The resolution of the survey scan was 120 000 (400 m/z) with a target value of 1×10^6^ ions, one microscan and maximum injection time of 200 ms. Low resolution CID MS/MS spectra were acquired with a target value of 10 000 in rapid CID scan mode with m/z range adjusted according to actual precursor mass and charge. MS/MS acquisition in the linear ion trap was carried out in parallel to the survey scan in the Orbitrap analyser by using the preview mode. The maximum injection time for MS/MS was 150 ms. Dynamic exclusion was enabled for 45 s after one MS/MS spectra acquisition and early expiration was disabled. The isolation window for MS/MS fragmentation was set to 2 m/z.

The analysis of the mass spectrometric RAW data files was carried out using the Proteome Discoverer software (Thermo Fisher Scientific; version 1.3) with in-house Mascot (Matrixscience, London, UK; version 2.4.1) and Sequest search engines utilisation. MS/MS ion searches were done against UniProt protein database for human (downloaded from ftp://ftp.uniprot.org/pub/databases/uniprot/current_release/knowledgebase/proteomes/; version 20130501; 88,817 sequences; 35,203,205 residues) with additional sequences from cRAP database (downloaded from http://www.thegpm.org/crap/). Mass tolerance for peptides and MS/MS fragments were 5 ppm and 0.5 Da, respectively. Oxidation of methionine and deamidation (N, Q) as optional modification, carbamidomethylation of C as fixed modification and two enzyme miss cleavages were set for all searches. Percolator was used for post-processing of Mascot and Sequest search results. Peptides with false discovery rate (FDR; q-value) < 1%, rank 1 and with at least 6 amino acids were considered. Label-free quantification using protein area calculation in Proteome Discoverer was used (“top 3 protein quantification” (6)).

Two LC-MS/MS analyses in total were done for each sample with the same sample volume. The second LC-MS/MS analysis was performed with exclusion of m/z masses already assigned to peptide from target database (FDR < 1%) based on the first LC-MS/MS analysis. Mass tolerance for m/z exclusion was set to 10 ppm and retention time window to 2 min. The two resulting raw files for each sample were searched as single data set.

**Analysis of the tetramer and pentamer motifs distribution in genomic regions encoding snRNAs**

All data analysis and plotting was performed using statistical software package R (http://www.r-project.org). The database of snRNAs has been created using the R-package biomaRt (7,8). All non-overlapping snRNAs annotated in Ensembl gene model (9), release 74, dataset “Homo sapiens genes”, version GRCh37.p13, were collected and analyzed (1916 records in total). The occurrence of oligonucleotide-stretches was treated using R-package Biostrings.

**Supplementary references**

1. Lubas, M., Christensen, M.S., Kristiansen, M.S., Domanski, M., Falkenby, L.G., Lykke-Andersen, S., Andersen, J.S., Dziembowski, A. and Jensen, T.H. (2011) Interaction profiling identifies the human nuclear exosome targeting complex. *Mol Cell*, **43**, 624-637.

2. Sanudo, M., Jacko, M., Rammelt, C., Vanacova, S. and Stefl, R. (2011) 1H, 13C, and 15N chemical shift assignments of ZCCHC9. *Biomol NMR Assign*, **5**, 19-21.

3. Wisniewski, J.R., Ostasiewicz, P. and Mann, M. (2011) High recovery FASP applied to the proteomic analysis of microdissected formalin fixed paraffin embedded cancer tissues retrieves known colon cancer markers. *J Proteome Res*, **10**, 3040-3049.

4. Wisniewski, J.R., Zougman, A., Nagaraj, N. and Mann, M. (2009) Universal sample preparation method for proteome analysis. *Nat Methods*, **6**, 359-362.

5. Stejskal, K., Potesil, D. and Zdrahal, Z. (2013) Suppression of peptide sample losses in autosampler vials. *J Proteome Res*, **12**, 3057-3062.

6. Silva, J.C., Gorenstein, M.V., Li, G.Z., Vissers, J.P. and Geromanos, S.J. (2006) Absolute quantification of proteins by LCMSE: a virtue of parallel MS acquisition. *Mol Cell Proteomics*, **5**, 144-156.

7. Durinck, S., Moreau, Y., Kasprzyk, A., Davis, S., De Moor, B., Brazma, A. and Huber, W. (2005) BioMart and Bioconductor: a powerful link between biological databases and microarray data analysis. *Bioinformatics*, **21**, 3439-3440.

8. Durinck, S., Spellman, P.T., Birney, E. and Huber, W. (2009) Mapping identifiers for the integration of genomic datasets with the R/Bioconductor package biomaRt. *Nature protocols*, **4**, 1184-1191.

9. Flicek, P., Amode, M.R., Barrell, D., Beal, K., Billis, K., Brent, S., Carvalho-Silva, D., Clapham, P., Coates, G., Fitzgerald, S. *et al.* (2014) Ensembl 2014. *Nucleic Acids Res*, **42**, D749-755.
